# New Implant Design with Midcrestal and Apical Wing Thread for Increased Implant Stability in Single Postextraction Maxillary Implant

**DOI:** 10.1155/2019/9529248

**Published:** 2019-09-05

**Authors:** Antonio Scarano, Bartolomeo Assenza, Francesco Inchingolo, Filiberto Mastrangelo, Felice Lorusso

**Affiliations:** ^1^Department of Medical, Oral and Biotechnological Sciences and CeSi-MeT, University of Chieti-Pescara, Via dei Vestini, 31, 66100 Chieti, Italy; ^2^Zirconia Implant Research Group (Z.I.R.G), International Academy of Ceramic Implantology, USA; ^3^Department of Oral Implantology, Dental Research Division, College Ingà, UNINGÁ, Cachoeiro de Itapemirim 29312, Brazil; ^4^Department of Interdisciplinary Medicine, University of Bari “Aldo Moro”, 70121 Bari, Italy; ^5^Department of Clinical and Experimental Medicine, University of Foggia, Foggia, Italy

## Abstract

**Background:**

The immediate placement of a dental implant could represent an option treatment for the rehabilitation of a postextractive missing tooth socket to replace compromised or untreatable teeth, with the advantage of single-session surgery. In this way, the anatomy of the alveolar bone defect, the preservation of the buccal cortical bone, and the primary stability of the fixture represent the critical factors that consent a precise implant placement.

**Objective:**

This case report describes a novel fixture design for postextractive alveolar socket immediate implant.

**Methods:**

Two patients (25 and 31 years old) were treated for postextractive dental implant placement to replace both central upper incisor teeth with four implants. The residual bone implant gap was not filled with graft or bone substitute. The restoration was provided following a standard loading protocol by a cement-sealed prosthetic abutment.

**Results:**

Clinically, all implants positioned showed an excellent insertion torque. No postoperative complications were reported. At 6 months of healing, the buccal cortical bone and the implant stability were present and well maintained.

**Conclusion:**

The evidence of this study allows us to underline the possible advantages of this new fixture design for postextractive implant technique.

## 1. Introduction

Nowadays, dental implants are recognized as a reliable treatment option for replacing missing teeth for periodontal defects, endodontic problems, trauma, fracture, and bone tissue atrophy is often present, which is more marked in a horizontal direction [1, 2].

The presence of vertical and horizontal lack of bone tissue represents an indispensable condition in order to reach stable implant-prosthetic rehabilitation over time [3–5]. Primary implant stability is a key factor that influences the success rate of these implants and determines the timing of prosthetic loading [6]. Many factors influence implant stability such as quality and quantity [7], shape, size, length, and the variety of surfaces [8, 9]. Primary implant stability is also important in case of immediate implant placement into fresh extraction sockets [10].

In this clinical situation, there is a bone defect and primary implant stability is very difficult to achieve. The surgical method allows reaching aspired aesthetical results, but a certain failure percentage has been reported [11, 12] due to different causes such as the presence of bacteria, absence of good primary stability, and lack of adequate bone support. Nowadays, the most used surgical protocol constitutes removing the causal element and waiting 2 months or more in order to reach socket healing. In a case of inadequate bone tissue where regenerative surgery is used to rehabilitate function and aesthetics [12, 13], it is necessary to wait an additional healing period.

In case of immediate postextractive implants for increasing the implant stability, a new implant geometry was introduced, aimed specifically at enabling better initial and long-term stability. This implant has a specially designed expanded diameter and a midcrestal and apical “wing” thread, which provides added bone contact for a higher insertion torque for primary stability.

The aim of this case report was to evaluate this novel implant design in two patients with immediate postextractive implant in an aesthetic area.

## 2. Case Report

A total of two patients, a 25-year-old male and a 31-year-old female, who required extraction of both central upper incisor teeth because of caries were selected. Neither patient presented chronicle or acute diseases that could influence osseointegration. The patients were nonsmokers with a good and correct domiciliary oral hygiene. A three-dimensional radiographic tomography scan (CBCT) (Vatech Ipax 3D PCH-6500, Fort Lee, NJ, USA) was performed before and after 6 months of implant placement. Two grams of amoxicillin was prescribed to both patients 2 hours before surgery. Chlorhexidine digluconate mouthwashes (Curaden Healthcare S.p.A., Saronno, Italy) were also recommended for at least 7 days after every meal or beverage, avoiding rinsing by water.

**Figure 1:**
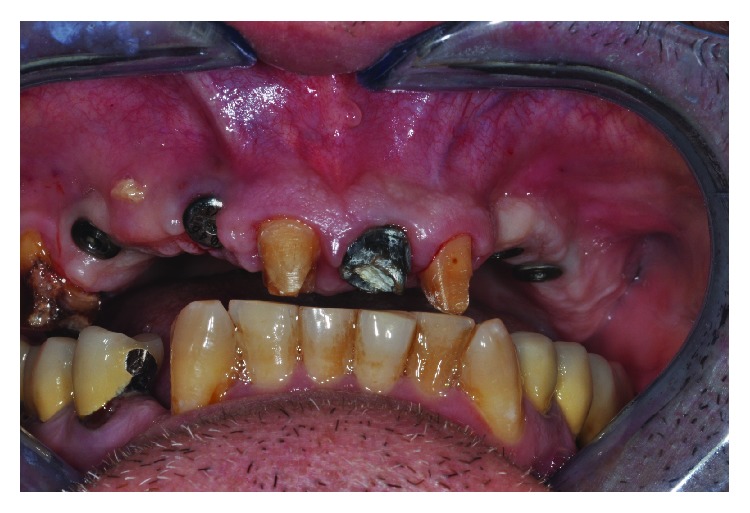
The teeth before extraction.

**Figure 2:**
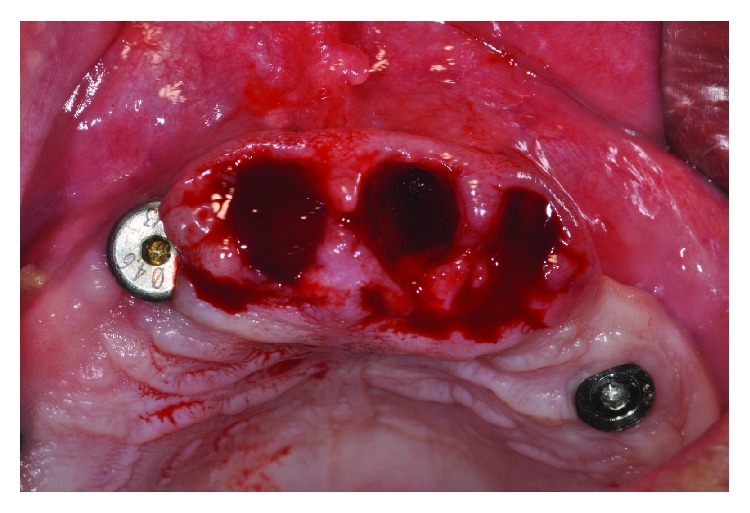
Extraction of the compromised element without flap elevation.

**Figure 3:**
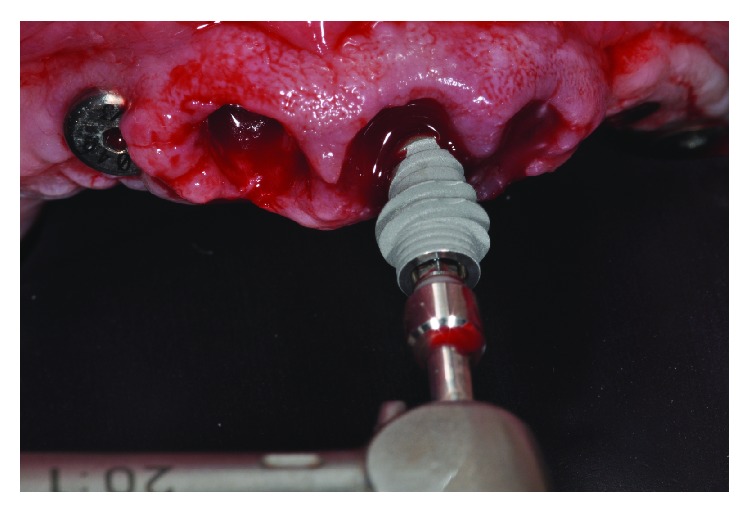
Placement of the implant with expanded diameter thread in the midcrestal and apical area.

The extraction of the teeth was executed without flap in order to allow postextractive implant placement (Figures 1 and 2). To prepare the implant site, a 2 mm diameter bur was first used that was 15° palatally inclined and worked along the socket remaining after extraction; this inclination allows the palatal direction preserving greater quantity of vestibular bone tissue. It must be emphasized that the implant sites did not respect socket length completely, since they had to move apically in a palatal direction. Compared to the implant, the site has to have a smaller section (0.5 mm) in order to guarantee greater quantity of vestibular bone tissue and assure valid primary stability. The final bur length has to be equal to the inserting fixture, which was 12 mm in these two cases. The implant site was prepared with dedicated surgical cutters, as indicated by the manufacturer with a sequential cutter passage: pilot cutter 2 mm and 3.1 mm, 3.9 mm then 4.25 mm terminal cutter. To verify the height of the implant site preparation, a 2 mm diameter and 12 mm length measurer was inserted. The insertion of a 4.5 mm diameter and 12 mm length X-Space implant (Bone System® Implant System, Milano, Italy) completed the surgery (Figure 3). This dental implant has a rounded apex and two sharp and highly engaging threads, located close to each other to facilitate implant insertion.

The implants were placed with a mean insertion torque of 35.5 ± 4 Ncm measured with a manual torque wrench by the dentist and recorded for statistical analysis (GraphPad 6.0, Prism, San Diego, USA). No bone substitute was used for filling the implant gap. The implants were loaded with standard protocol, and the reverse torque was evaluated at 6 months after healing in the second phase surgery [14, 15].

## 3. Results

**Figure 4:**
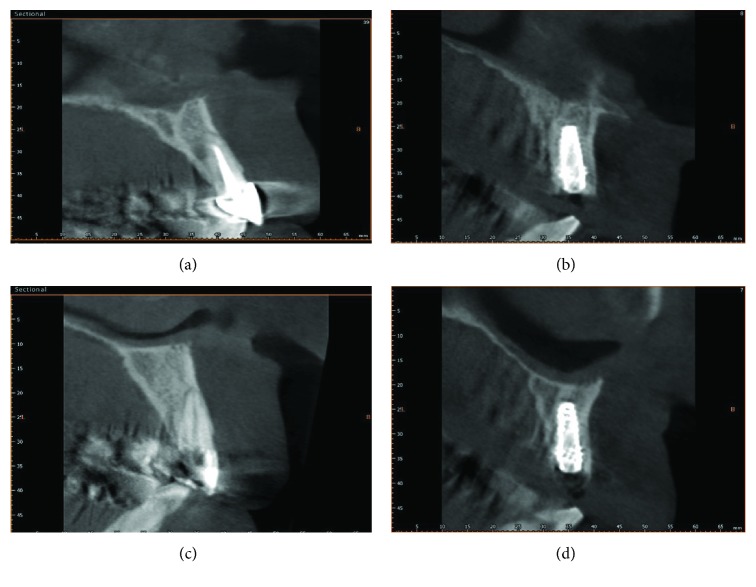
(a) Before extraction the CBCT image showing a thin buccal plate. (b) After 6 months of implant placement, the buccal plate was preserved and the gap was filled by new a bone. (c) Second clinical case. Before extraction, there is a thin buccal plate. (d) After 6 months of implant placement. Also in this case, the buccal plate was preserved.

The four implant implants showed good radiologic osseointegration (Figure 4). The application of a 60 Ncm torque by a manual torque after 6 months of implant placement was not sufficient for detaching them from the bone. This confirms the good integration of the implants. A three-dimensional radiographic tomography scan (CBCT) (Vatech Ipax 3D PCH-6500, Fort Lee, NJ, USA) was performed before the procedure. The CBCT image after 6 months of implant placement showing the preserved buccal plate and the gap was filled by a new bone (Figure 4).

## 4. Discussion

The application of this new implant design in immediate postextractive socket guarantees a valid primary stability that is indispensable for following implant osseointegration. The use of the studied implant with an expanded diameter and midcrestal and apical “wing” thread, inserted by dynamometer ratchet pressure, determines preservation of the postextractive site and increases implant stability. Immediate implant placement into fresh extraction sockets is extensively used in clinical practice and is considered a predictable and acceptable procedure [16, 17]. In this report, the procedure for immediate implant placement with an expanded diameter thread in the midcrestal and apical area was conducted as planned and a favourable result was obtained, as shown by the initial postoperation images. Ever since the immediate implant placement procedure was developed, the maintenance of the buccal bony wall and implant stability has always been a major concern for implantologists.

## 5. Conclusion

After 6 months of clinical observation, this report demonstrates that the stability of immediately placed implants can be ensured by an expanded diameter thread in the midcrestal and apical area. However, due to the limitations associated with the use of only two case reports and four implants, further research is required to confirm it.
